# Coccidian parasites in the endangered Forest Musk Deer (*Moschus berezovskii*) in China, with the description of six new species of *Eimeria* (Apicomplexa: Eimeriidae)

**DOI:** 10.1051/parasite/2021067

**Published:** 2021-10-19

**Authors:** Yunyun Gao, Donald W. Duszynski, Fulin Yuan, Defu Hu, Dong Zhang

**Affiliations:** 1 Key Laboratory of Non-Invasive Research Technology for Endangered Species, School of Nature Conservation, Beijing Forestry University Beijing 10083 PR China; 2 Department of Zoology, School of Ecology and Nature Conservation, Beijing Forestry University 35 Qinghua East Road Beijing 100083 China; 3 Emeritus Professor of Biology 76 Homesteads Rd. Placitas NM 87043 USA

**Keywords:** Coccidia, *Eimeria*, New species, Prevalence, *Moschus berezovskii*

## Abstract

We examined 674 fresh fecal samples from forest musk deer (*Moschus berezovskii* Flerov) in Sichuan and Shaanxi Provinces, China, for coccidian oocysts and 65% were infected with *Eimeria* spp. Previously, only four *Eimeria* species were known from *Moschus* spp. Here we describe six new *Eimeria* species. *Eimeria aquae* n. sp., in 38% deer, has ovoidal oocysts, 32.0 × 23.0 μm, micropyle (M) and scattered polar granules (PGs) of various sizes are present, sometimes oocyst residuum (OR) is present; ovoidal sporocysts, 14.1 × 7.5 μm, with Stieda body (SB) and sporocyst residuum (SR). *Eimeria dolichocystis* n. sp., in 11% deer; cylindroidal oocysts, 36.6 × 18.9, with a M, 1 PG and OR; ovoidal sporocysts, 13.9 × 7.7, with SB and SR. *Eimeria fengxianensis* n. sp., in 7% deer; ovoidal oocysts, 36.3 × 25.2, a M and PGs present but OR absent; ovoidal sporocysts, 13.9 × 7.3, with SB and SR. *Eimeria helini* n. sp. in 24% deer; subspheroidal oocysts, 27.0 × 24.1, OR and PGs often present, but M absent; ovoidal sporocysts, 13.5 × 7.7, with SB and SR. *Eimeria kaii* n. sp. in 26% deer; ovoidal oocysts, 33.2 × 20.7, M and PGs present, but OR absent; ovoidal sporocysts, 14.4 × 7.5, with SB and SR. *Eimeria oocylindrica* n. sp., in 17% deer; cylindroidal oocysts, 36.0 × 21.4, M and 1-2 PGs present but OR absent; ovoidal sporocysts, 13.8 × 7.7, with SB and SR. *Eimeria dujiangyanensis* n. nom. is proposed to replace *E. moschus* Sha, Zhang, Cai, Wang & Liu, 1994, a junior homonym of *E. moschus* Matschoulsky, 1947.

## Abstract (fr)

Nous avons examiné 674 échantillons de matières fécales fraîches de cerf porte-musc (*Moschus berezovskii* Flerov) dans les provinces du Sichuan et du Shaanxi, en Chine, pour les oocystes de coccidies et 65 % étaient infectés par *Eimeria* spp. Auparavant, seules quatre espèces d’*Eimeria* étaient connues chez *Moschus* spp. Nous décrivons ici six nouvelles espèces d’*Eimeria*. *Eimeria aquae* n. sp., chez 38 % des cerfs, a des oocystes ovoïdes, 32,0 × 23,0 μm, micropyle (M) et granules polaires (PG) de différentes tailles présents, parfois des résidus d’oocystes (OR) présents; sporocystes ovoïdes, 14,1 × 7,5 μm, avec corps de Stieda (SB) et résidu de sporocyste (SR). *Eimeria dolichocystis* n. sp., chez 11 % des cerfs; oocystes cylindroïdes, 36,6 × 18,9, avec un M, 1 PG et OR; sporocystes ovoïdes, 13,9 × 7,7, avec SB et SR. *Eimeria fengxianensis* n. sp., chez 7 % des cerfs; oocystes ovoïdes, 36,3 × 25,2, M et PG présents mais OR absent; sporocystes ovoïdes, 13,9 × 7,3, avec SB et SR. *Eimeria helini* n. sp. chez 24 % des cerfs; oocystes subsphéroïdaux, 27,0 × 24,1, OR et PG souvent présents, mais M absent; sporocystes ovoïdes, 13,5 × 7,7, avec SB et SR. *Eimeria kaii* n. sp. chez 26 % des cerfs; oocystes ovoïdes, 33,2 × 20,7, M et PG présents, mais OR absent; sporocystes ovoïdes, 14,4 × 7,5, avec SB et SR. *Eimeria oocylindrica* n. sp., chez 17 % des cerfs; oocystes cylindroïdes, 36,0 × 21,4, M et 1-2 PG présents mais OR absent; sporocystes ovoïdes, 13,8 × 7,7, avec SB et SR. *Eimeria dujiangyanensis* n. nom. est proposé pour remplacer *E. moschus* Sha, Zhang, Cai, Wang & Liu, 1994, homonyme junior de *E. moschus* Matschoulsky, 1947.

## Introduction

*Moschus* is the only genus in the family Moschidae, with seven extant species, which are all called musk deer [6, 16, 21]. Although they resemble small deer, they are not true deer (Cervidae) because they lack the antlers and facial glands of deer and because they possess only one pair of teats, a gall bladder, a musk gland in males, and a pair of tusk-like teeth that cervids lack [20, 24]. These small deer live in hilly scrub and forested habitat in the mountains of Asia (notably the Himalayas). They are solitary, shy herbivores with well-defined territories and are either crepuscular or nocturnal. Small populations are spread through many Asian countries (Afghanistan, Bhutan, Burma, China, India, Korea, Mongolia, Nepal, Pakistan, Russia [southeastern Siberia], Tibet, and Vietnam) where most species and populations are either declining, endangered or threatened because of poaching for their musk glands (cosmetics, perfumes) and/or because of excessive hunting for illegal trade and habitat loss due to deforestation [1, 20, 24, 26].

*Moschus berezovskii* Flerov, 1929, the Chinese forest musk deer, is distributed mostly in China where it is classified as “Endangered” by the International Union for Conservation of Nature (IUCN). Unfortunately, its populations were declining rapidly due to exploitation and extensive habitat loss [18, 23, 25] until a captive breeding program for *M. berezovskii* began in China in 1958 [7, 8, 20, 24] and from that effort, 13 *M. berezovskii* (8 females, 5 males) were reintroduced back into the wild in 2017 (https://www.sohu.com/a/153114346_157267).

Gastrointestinal parasitism by microbes, protists and helminths is common in these musk deer and may lead to > 30% mortality rates in captive musk deer [7, 8, 13] which have seriously hampered their population expansion. Based on several surveys of gastrointestinal parasitism of forest musk deer, oocysts of *Eimeria* spp. were the dominant parasite group observed, with high prevalence, and obvious differences of parasite infection in both musk deer sex and age groups [2, 12, 14]. In the wild, forest musk deer are extremely cryptic, and detailed accounts for gastrointestinal parasites in all age groups under natural conditions are lacking. Thus, captive animals provide an excellent opportunity to study and understand the parasite communities of wildlife and, in our work, especially the Chinese musk deer on 13 captive breeding centers in China.

Here we summarized our current knowledge of the morphology and taxonomy on the *Eimeria* species reported to date from *Moschus* species, described six new *Eimeria* species, and corrected the nomenclature of a previously named species based on scientific priority. We also evaluated the differences of the various *Eimeria* species between adult and juvenile musk deer.

## Materials and methods

### Ethics approval

Our fecal sample collections in this study were done with the approval of the School of Ecology and Nature Conservation, Beijing Forestry University (EAWC_BJFU_2021008), under the guidelines of the Institution of Animal Care and the Ethics Committee of Beijing Forestry University, and the help of local veterinarians.

### Host and location information

From August to September 2020, fresh fecal samples were collected from 674 forest musk deer in 13 captive breeding centers of forest musk deer in Sichuan and Shaanxi Provinces; these centers include more than 80% of all captive centers in China. All breeding centers have similar breeding systems, with a male and four or five females kept in the same enclosure, and each individual with ear tagging in adult and collar in young, has an independent brick cell as described in Hu et al. [7]. The staff of the breeding centers clean each cell every day, thus allowing the collection of fresh feces from each musk deer. In addition, all centers are located in the mountains at an altitude of 1100–1500 m close to the Qinling Mountains and Tibetan Plateau, an area that is incredibly rich in biodiversity. Such regions provide suitable conditions of climate and natural food for forest musk deer and, thus, are ideal for breeding centers. Eight breeding centers are located in Shaanxi Province, a region of the southern Qinling Mountains, with six in Baoji (33°45′ N, 106°40′ E) and two in Hanzhong (33°35′ N, 106°49′ E). Five breeding centers are located in Sichuan province, a region on the eastern Tibetan Plateau, with two in Aba (31°24′ N, 103°14′ E) and three in Guangyuan (32°37′ N, 104°45′ E).

### Morphological analysis

A total of 674 fresh fecal samples were collected, then each sample was crushed and mixed. Each sample of 5 g mixed feces was placed in 40 mL screw-cap plastic vials containing 20 mL of 2.5% (w/v) aqueous potassium dichromate (K_2_Cr_2_O_7_) solution. Fecal suspensions were then spread out in Petri dishes and incubated at 25 °C for 2 weeks, allowing time for oocyst sporulation. Following the guidelines of Duszynski & Wilber [4], we examined oocysts by coverslip flotation in Sheather’s sugar solution (S.G. = 1.20), after which they were measured and photographed. Morphological observation and measurements were obtained using oil immersion objectives (Zeiss AX10, Germany), a Nomarski contrast microscope (Zeiss Axio scope 5, Germany) and a Confocal Laser Scanning microscope (Leica DMI 6000 CS, Germany). All measurements (in μm) are presented as the means followed by their ranges in parentheses, and the oocysts and sporocysts are described following the guidelines of Duszynski & Wilber [4]. For the calculation, a 95% confidence interval was used. The prevalence rate, statistical significance were analyzed using Prism GraphPad Prism software (GraphPad, San Diego, CA, USA) as described in Hua et al. [9]. In all cases, *p* values less than 0.05 were considered significant.

### Abbreviation

As has become standard practice in oocyst descriptions, we use the abbreviations of Duszynski and Wilber [4] in all our species descriptions: *L*, length; *W*, width; *L*/*W*, length/width ratio (shape index); M, micropyle; MC, micropyle cap; PG, polar granule; OR, oocyst residuum; RB, refractile body in sporozoite; SB, Stieda body in sporocyst; SSB, substieda body; PSB: parastieda body; SR, sporocyst residuum; SZ, sporozoite; N, nucleus of SZ; 95% CI: 95% confidence interval.

## Results

Out of 674 fecal samples evaluated, 226 were from females, 263 from males, and 185 from young individuals. Oocysts that we detected represent six new *Eimeria* spp. and one or more species were found in 441 or 65% samples (95% CI: 0.62, 0.69) with no significant difference (*p* = 0.85, 95% CI: −0.08, 0.12) between males (57%, 95% CI: 0.51, 0.63) and females (59%, 95% CI: 0.53, 0.66). However, the prevalence of *Eimeria* in young individuals (85%, 95% CI: 0.80, 0.90) was significantly higher than males (*p* < 0.01, 95% CI: −0.38, −0.17) and females (*p* < 0.01, 95% CI: −0.36, −0.15). Of the positive fecal samples, 195/441 (44%) were found to harbor only one *Eimeria* species at the time of sampling, 146/441 (33%) were infected with two *Eimeria* species, 73/441 (17%) had three species, 23/441 (5%) had four species, and 4/441 (1%) were concurrently infected with five or more *Eimeria* species.

In the following lines, we describe six new species from *M. berezovskii* in China.

### *Eimeria aquae* n. sp. (Figs. 1A, 1B; Fig. 2A)

urn:lsid:zoobank.org:act:AC05A343-493E-4021-964C-9440DAFF11EC

**Figure 1.**
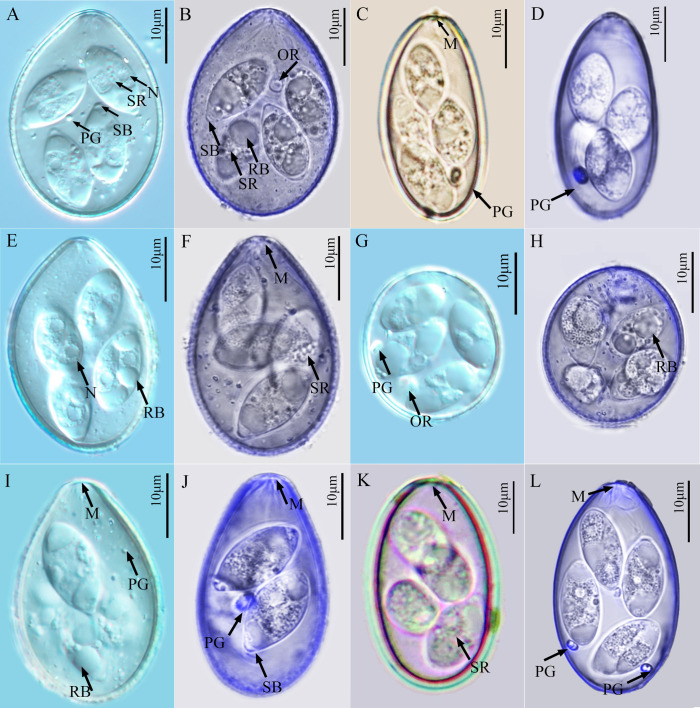
Photomicrographs of the sporulated oocysts of six *Eimeria* species collected from forest musk deer. A, B. *Eimeria aquae* n. sp.; C, D. *Eimeria dolichocystis* n. sp.; E, F. *Eimeria fengxianensis* n. sp.; G, H. *Eimeria helini* n. sp.; I, J. *Eimeria kaii* n. sp.; K, L. *Eimeria oocylindrica* n. sp.; Figs. A, E, G, I were obtained by differential interference contrast microscopy; Figs. B, D, F, H, J, L were obtained by confocal laser scanning microscopy; Figs. C, K were obtained under standard light microscopy under oil immersion. *Abbreviations*: M, micropyle; PG, polar granule; OR, oocyst residuum; RB, refractile body in sporozoite; SB, Stieda body on sporocyst; SR, sporocyst residuum; N, nucleus of SZ.

**Figure 2.**
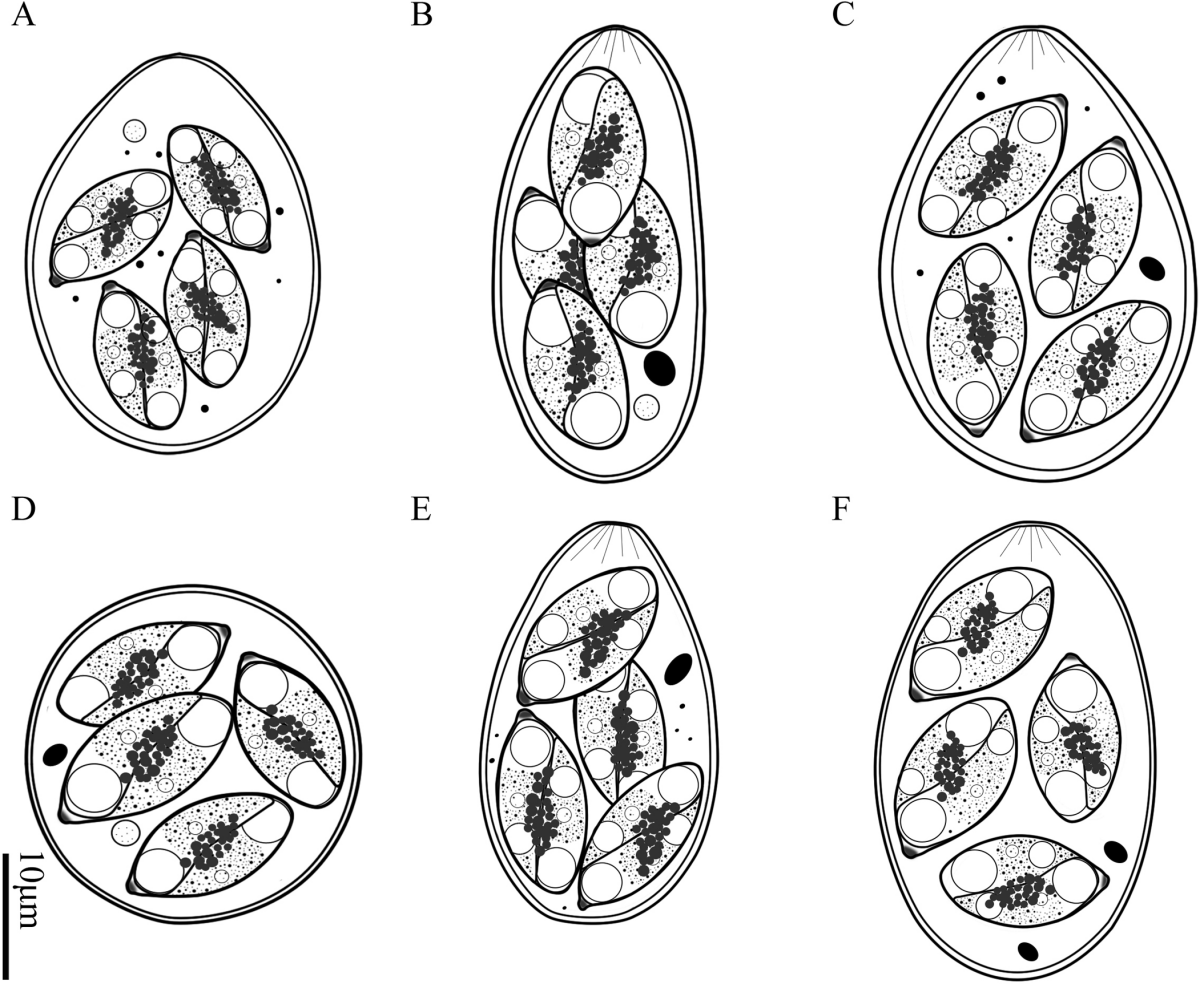
Line drawings of the sporulated oocysts of the six *Eimeria* species discovered and described from forest musk deer. A. *Eimeria aquae* n. sp.; B. *Eimeria dolichocystis* n. sp.; C. *Eimeria fengxianensis* n. sp.; D. *Eimeria helini* n. sp.; E. *Eimeria kaii* n. sp.; F. *Eimeria oocylindrica* n. sp.

*Type host:* Artiodactyla: Moschidae: *Moschus berezovskii* Flerov, 1929, Chinese forest musk deer.

*Type locality:* Hanzhong (33°35′ N, 106°49′ E), Shaanxi Province, China.

*Other hosts:* None to date.

*Description of sporulated oocyst:* Oocyst shape: ovoid; *L* × *W* (*n* = 64): 32.0 × 23.0 (27–33 × 21–26), *L*/*W* ratio: 1.3 (1.2–1.4); oocyst wall smooth, colourless to pale yellow, ~1.2 thick, bi-layered; oocyst wall thickest on sides and thinnest at end opposite M; MC: absent; M: present (*n* = 54), 3.0 (0.7–5.8) wide; PG: present as scattered granules of various sizes, 0.5–1.1 wide; OR: present in some oocysts (*n* = 7): ~2.0 (0.5–3.4) wide (Fig. 1B).

*Description of sporocyst and sporozoites:* Sporocyst shape: elongate-ovoidal; *L* × *W* (*n* = 62): 14.1 × 7.5 (10–16 × 5–9); *L*/*W* ratio: 1.9 (1.5–2.2); SB present; SSB, PSB: both absent; SR: present as small granules disbursed between SZ; SZ banana-shaped with one large RB at its more rounded end, and a second smaller RB in the anterior (more pointed) end; N, when visible, in middle of SZ between RBs.

*Prevalence:* Oocysts of this morphotype were found in 255/674 (38%) *M. berezovskii*, being found in 92/185 (50%) juveniles, in 90/263 (34%) adult males, and in 73/226 (32%) adult females.

*Sporulation:* Exogenous. Oocysts were shed unsporulated and became completely sporulated after two weeks in 2.5% (w/v) potassium dichromate solution (K_2_Cr_2_O_7_).

*Prepatent and patent periods*: Unknown.

*Site of infection:* Unknown, oocysts were recovered from feces after the animals defecated.

*Pathogeny:* Unknown.

*Materials deposited:* Photosyntypes [5] of sporulated oocysts are deposited in the Key Laboratory of Non-Invasive Research Technology for Endangered Species, School of Nature Conservation, Beijing Forestry University, Beijing, China, repository number is BFU-E-2, 2020.

*Etymology:* The species name is derived from the oocysts shape, which resembles a drop of water, and aqua- (L., water, water-like).

*Remarks:* The oocyst (Table 1) and sporocyst (Table 2) structural data of the other eimerians known to infect *Moschus* species worldwide, show that sporulated oocysts of *E. aquae* somewhat resemble those of a morphotype described by Sha et al. [19], which they called *E. moschus* (now *E. dujiangyanensis* N. Comb., see below), but the width of their M, size of their sporocysts, and shape of their SZs are all different. The length of *E. aquae* oocysts is similar to that of *E. kaii* but the width of the latter is much smaller resulting in distinctly different *L*/*W* ratios (1.3 vs. 1.5), and the number, size and shape of their PGs are different.

**Table 1.** Defining structural characters of the sporulated oocysts of all *Eimeria* species known from all seven musk deer species (Moschidae: *Moschus* spp.) worldwide.

Host sp./*Eimeria* sp.	Shape^1^	Wall^2^	*L* × *W* (range)	*L* × *W* (ratio)	Micropyle^3^(width)	Micropyle Cap (size)	Residuum (width)	Polar Granule/# (size)
*M. berezovskii*								
*E. aquae*	O	S/2	32.0 × 23.0	1.3	+	–	+/− (0.5–3.4)	+/a few
		(1.2)	(27–33 × 21–26)		(0.7–5.8)			(0.5–1.1)
*E. dolichocystis*	C/E	S/2	36.6 × 18.9	2.0	+	–	+/–	+/1–2
		(1.3)	(29–40 × 16–20)		(0.9–4.0)		(1.2–1.5)	(1.6–1.9)
*E. fengxianensis*	O	S/2	36.3 × 25.2	1.4	+	–	–	+/a few or 1
		(1.6)	(35–39 × 24–27)		(0.9–5.1)			(0.6–1.0)
*E. helini*	S/SS	S/2	27.0 × 24.1	1.1	–	–	+	+/1
		(1.1)	(20–30 × 16–27)				(0.3–3.0)	(1.9–2.6)
*E. jinfengshanenisis*	O	S/2	28.1 × 18.3	1.6	+	–	–	–
		(0.5–0.8)	(25–32.5 × 17.5–18.5)		(Inc.)			
*E. dujiangyanensis*	O	S/2	35.2 × 26.9	1.3	+	–	–	–
			(32.5–37.5 × 25–30)		(6–7)			
*E. kaii*	O	S/2	33.2 × 20.7	1.5	+	–	–	+/a few or 1–2
		(1.2)	(32–37 × 19–24)		(0.9–4.9)			(0.6–2.8)
*E. oocylindrica*	C/E/O	S/2	36.0 × 21.4	1.7	+	–	–	+/1–2
		(1.3)	(33–41 × 19–26)		(0.8–6.7)			(1.6–2.0)
*M. moschiferus*								
*E. moschus*	O	S/2	27.4 × 20.9	1.3	+	–	–	–
			(20–31 × 15–23)		(6.6)			
*E. sajanica*	S/SS	S/2	20.7 × 18.3	1.1	–	–	–	–
			(18–23 × 16.5–20)					

1 C = Cylindroidal; E = Ellipsoidal; O = Ovoidal (egg-shaped); S = Spheroidal (round); SS = Subspheroidal.

2 Oocyst wall characteristics: S = Smooth or R = Rough/No. of layers (thickness).

3 Inc. = Micropyle is present but inconspicuous.

**Table 2.** Defining structural characters of the sporocysts/sporozoites of all *Eimeria* species known from all seven musk deer species (Moschidae: *Moschus* spp.) worldwide.

Host sp./*Eimeria* sp.	Shape^1^	*L* × *W* (range)	*L* × *W* (ratio)	Stieda/Substieda/Parastieda bodies	Residuum (type/size)^2^	Refractile Bodies (No.)^3^	SZ Shape	SZ N^4^
*M. berezovskii*								
*E. aquae*	E/O	14.1 × 7.5	1.9	+/–/–	+	+	Banana-like	+
		(10–16 × 5–9)	(1.5–2.2)		(SG)	(2, L, S)		C
*E. dolichocystis*	E/O	13.9 × 7.7	1.8	+/–/–	+	+	Sausage-like	+/–
		(10–18 × 5–12)	(1.4–2.6)		(SG)	(1, L)		C
*E. fengxianensis*	E/O	13.9 × 7.3	1.9	+/–/–	+	+	Sausage-like	+/–
		(11–16 × 6–9)	(1.6–2.2)		(SG)	(2, L, S)		C
*E. helini*	E/O	13.5 × 7.7	1.8	+/–/–	+	+	Sausage-like	+/–
		(9–16 × 6–10)	(1.4–2.0)		(SG)	(1, L)		C
*E. jinfengshanenisis*	E	11.1 × 5.9	1.9	–/–/–	+	+	Banana-like	–
		(10–11 × 5–7)	(1.7–2.0)		(SG)	(1, Sm)		
*E. dujiangyanensis*	S	17.5 × 8.2	2.1	–/–/–	+	+	Sausage-like	–
		(16–20 × 7.5–10)	(1.9–2.3)		(SG)	(1, L)		
*E. kaii*	E/O	14.4 × 7.5	1.9	+/–/–	+	+	Banana-like	+/–
		(12–16 × 6–8)	(1.6–2.2)		(SG)	(2, L, S)		C
*E. oocylindrica*	E/O	13.8 × 7.7	1.8	+/–/–	+	+	Sausage-like	+/–
		(11–16 × 6–9.5)	(1.5–2.2)		(SG)	(2, L, S)		C
*M. moschiferus*								
*E. moschus*	E	Means not given	Not given	–/–/–	+	+	Banana-like	–
		(10–10.5 × 5–7)			(SG)	(1, Sm)		
*E. sajanica*	E	Means not given	1.1	–/–/–	+	+	Banana-like	–
		(5–10 × 3–5)			(SG)	(1, Sm)		

1 E = Ellipsoidal; E/O = Elongate-ovoidal (1 end rounded, 1 end pointed slightly); S = Spindle-shaped.

2 Sporocyst residuum: Shape, consistency (SG = scattered granules; CM = compact mass).

3 Refractile bodies of SZ: Number, general size (L = large at rounded end; S = smaller at pointed end; Sm = small at rounded end).

4 Sporozoite (SZ) nucleus (N): visible (+), not visible (–); location (C = central in SZ; W = wide end of SZ; P = pointed end of SZ).

### *Eimeria dolichocystis* n. sp. (Figs. 1C, 1D; Fig. 2B)

urn:lsid:zoobank.org:act:9F65AF5C-D2C5-48B8-BF75-D083D70839C0

*Type host:* Artiodactyla: Moschidae: *Moschus berezovskii* Flerov, 1929, Chinese forest musk deer.

*Type locality:* Guangyuan (32°37′ N, 104°45′ E), Sichuan Province, China.

*Other hosts:* Unknown, none to date.

*Description of sporulated oocyst:* Oocyst shape: elongated cylindroidal or, ellipsoidal; *L* × *W* (*n* = 23): 36.6 × 18.9 (29–40 × 16–20), *L*/*W* ratio: 2.0 (1.9–2.2); oocyst wall smooth and colorless to pale yellow, ~1.3 thick, bi-layered; oocyst wall thickest on sides and thinnest at end opposite the M; MC: absent; M: present (*n* = 13), ~2.3 (0.9–4.0) wide; PG: always one, but a few have two (1.6–1.9 wide); OR: present in some oocysts (*n* = 4), 1.3 (1.2–1.5) wide.

*Description of sporocyst and sporozoites:* Sporocyst shape: elongate ovoidal; *L* × *W* (*n* = 23): 13.9 × 7.7 (10–18 × 5–12); *L*/*W* ratio: 1.8 (1.4–2.6); SB present; SSB, PSB: both absent; SR: present as small granules disbursed between SZ; SZ: sausage-like with one large RB at rounded end of SZ, and sometimes a second, smaller RB in the anterior end; N of SZ, when visible, between the RBs.

*Prevalence:* Oocysts of this morphotype were found in 73/674 (11%) *M. berezovskii*, being found in 39/185 (21%) juveniles, in 15/263 (6%) adult males, and in 19/226 (8%) adult females.

*Sporulation:* Exogenous. Oocysts were shed unsporulated and become completely sporulated after two weeks in 2.5% (w/v) potassium dichromate solution (K_2_Cr_2_O_7_).

*Prepatent and patent periods*: Unknown.

*Site of infection:* Unknown, oocysts were recovered from feces after the animals defecated.

*Pathogeny:* Unknown.

*Materials deposited:* Photosyntypes [5] of sporulated oocysts are deposited in the Key Laboratory of Non-Invasive Research Technology for Endangered Species, School of Nature Conservation, Beijing Forestry University, Beijing, China, repository number is BFU-E-8, 2020.

*Etymology:* The species name is derived from the long oocyst shape using *dolichol-* (Gk., long).

*Remarks:* The elongated, cylindroidal shape of the oocysts of *E. dolichocystis* is its most distinctive feature and their *L*/*W* ratio is the largest of all known and previously described species (Table 1). This feature clearly distinguishes it from all other know eimerians in *Moschus*.

### *Eimeria fengxianensis* n. sp. (Figs. 1E, 1F; Fig. 2C)

urn:lsid:zoobank.org:act:D240F705-FB6A-4158-AA12-FCC0F4100778

*Type host:* Artiodactyla: Moschidae: *Moschus berezovskii* Flerov, 1929, Chinese forest musk deer.

*Type locality:* Baoji (33°45′ N, 106°40′ E), Shaanxi Province, China.

*Other hosts:* Unknown, none to date.

*Description of sporulated oocyst:* Oocyst shape: ovoidal; *L* × *W* (*n* = 31): 36.3 × 25.2 (35–39 × 24–27), *L*/*W* ratio: 1.4 (1.4–1.5); oocyst wall smooth and colourless to pale yellow, ~1.6 thick, bi-layered; oocyst wall thickest on sides and thinnest at end opposite M; MC: absent; M: present (*n* = 30), 2.1 (0.9–5.1) wide; PG: a few, scattered throughout oocyst; PG: present, 0.6–1.0 wide; OR: absent.

*Description of sporocyst and sporozoites:* Sporocyst shape: elongate-ovoidal; *L* × *W* (*n* = 27): 13.9 × 7.3 (11–16 × 6–9); *L*/*W* ratio: 1.9 (1.6–2.2); SB: present; SSB, PSB: both absent; SR: present as small granules disbursed between SZ; SZ: sausage-like, usually with one large RB at rounded end of SZ and a smaller RB near the more pointed end; N: when visible, in middle of SZ between RBs.

*Prevalence:* Oocysts of this morphotype were found in 45/674 (7%) *M. berezovskii*, being found in 19/185 (10%) juveniles, in 11/263 (4%) adult males, and in 15/226 (7%) adult females.

*Sporulation:* Exogenous. Oocysts were shed unsporulated and became completely sporulated after 2 weeks in 2.5% (w/v) potassium dichromate solution (K_2_Cr_2_O_7_).

*Prepatent and patent periods*: Unknown.

*Site of infection:* Unknown, oocysts were recovered from feces after the animals defecated.

*Pathogeny:* Unknown.

*Materials deposited:* Photosyntypes [5] of sporulated oocysts are deposited in the Key Laboratory of Non-Invasive Research Technology for Endangered Species, School of Nature Conservation, Beijing Forestry University, Beijing, China, repository number is BFU-E-6, 2020.

*Etymology:* Fengxian (Shaanxi Province, China) is the hometown of forest musk deer in China. The species name is derived from this town’s name and -ensis (L., belonging to). The name is to thank the local people for their efforts to protect the forest musk deer.

*Remarks:* The sporulated oocysts of *E. fengxianensis* are similar to those of *E. aquae* (Tables 1, 2); however, there are some subtle differences that we believe are constant and can help one distinguish between the two species. Sporulated oocysts of the former are larger than those of *E. aquae* (36 × 25 [35–39 × 24–27] vs. 32 × 23 [27–33 × 21–26]), they always have a much thicker oocyst wall (1.6 vs. 1.2), slightly larger *L*/*W* ratio (1.4 vs. 1.3), and they lack any remnant of either PG and/or OR, which some of the oocysts of *E. aquae* often display as 1, 2, or more spheroidal bodies (cf. Figs 1E, 1F vs. Figs 1A, 1B).

### *Eimeria helini* n. sp. (Figs. 1G, 1H; Fig. 2D)

urn:lsid:zoobank.org:act:7B58FD5A-2EE0-4FD7-AC09-4F73ED4ECB06

*Type host:* Artiodactyla: Moschidae: *Moschus berezovskii* Flerov, 1929, Chinese forest musk deer.

*Type locality:* Aba (31°24′ N, 103°14′ E), Sichuan Province, China.

*Other hosts:* Unknown, none to date.

*Description of sporulated oocyst:* Oocyst shape: spheroidal to subspheroidal; *L* × *W* (*n* = 9): 27.0 × 24.1 (20–30 × 16–27), *L*/*W* ratio: 1.1 (1–1.3); oocyst wall smooth and colorless to pale yellow, ~1.1 thick, bi-layered; oocyst wall that may thin slightly at one end; M, MC: both absent; PG: always one present (1.9–2.6 wide); OR: present (*n* = 2): 0.3–3.2 wide.

*Description of sporocyst and sporozoites:* Sporocyst shape: elongate ovoidal; *L* × *W* (*n* = 9): 13.5 × 7.7 (9–16 × 6–10); *L*/*W* ratio: 1.8 (1.4–2.0); SB present; SSB, PSB: both absent; SR: present as small granules disbursed between SZ; SZ: sausage-like, usually with one large RB at more rounded end of SZ and sometimes a second, smaller RB is seen in the more pointed end; N: when visible, in middle of SZ between RBs.

*Prevalence:* Oocysts of this morphotype were found in 159/674 (24%) *M. berezovskii*, being found in 53/185 (29%) juveniles, in 60/263 (23%) adult males, and in 46/226 (20%) adult females.

*Sporulation:* Exogenous. Oocysts were shed unsporulated and they completely sporulated after 2 weeks in 2.5% (w/v) potassium dichromate solution (K_2_Cr_2_O_7_).

*Prepatent and patent periods*: Unknown.

*Site of infection:* Unknown, oocysts were recovered from feces after the animals defecated.

*Pathogeny:* Unknown.

*Materials deposited:* Photosyntypes [5] of sporulated oocysts are deposited in the Key Laboratory of Non-Invasive Research Technology for Endangered Species, School of Nature Conservation, Beijing Forestry University, Beijing, China, repository number is BFU-E-9, 2020.

*Etymology:* The species name is given in honor of Helin Sheng, a retired professor at East China Normal University, who has been devoted to musk deer conservation and research for more than 30 years.

*Remarks:* The spheroidal to subspheroidal shape of the sporulated oocysts is a distinctive feature in *E. helini* that allows it to be distinguished from all other eimerians now known to infect *Moschus* species.

### *Eimeria kaii* n. sp. (Fig. 1I, 1J; Fig. 2E)

urn:lsid:zoobank.org:act:46620F9C-97ED-4B63-A16C-7E1B376EE6DC

*Type host:* Artiodactyla: Moschidae: *Moschus berezovskii* Flerov, 1929, Chinese forest musk deer.

*Type locality:* Baoji (33°45′ N, 106°40′ E), Shaanxi Province, China.

*Other localities:* Hanzhong (33°35′ N, 106°49′ E), Shaanxi Province, China. Aba (31°24′ N, 103°14′ E), Sichuan Province, China. Guangyuan (32°37′ N, 104°45′ E), Sichuan Province, China.

*Other hosts:* Unknown, none to date.

*Description of sporulated oocyst:* Oocyst shape: ovoidal; *L* × *W* (*n* = 94): 33.2 × 20.7 (32–37 × 19–24), *L*/*W* ratio: 1.5 (1.5–1.7); oocyst wall smooth and colourless to pale yellow, ~1.2 thick, bi-layered; oocyst wall thickest on sides and thinnest at end opposite the M; MC: absent; M: present (*n* = 81): ~1.9 (0.9–4.9) wide; PG: present only as a few scattered granules or 1–2 spheroidal bodies, 0.6–2.8 wide; OR: absent.

*Description of sporocyst and sporozoites:* Sporocyst shape: elongate-ovoidal; *L* × *W* (*n* = 92): 14.4 × 7.5 (12–16 × 6–8); *L*/*W* ratio: 1.9 (1.6–2.2); SB: present; SSB, PSB: both absent; SR: present as small granules disbursed between SZ; SZ: usually with one large RB at more rounded end of SZ, and a second smaller RB in the thinner end of the SZ; N: when visible, in middle of SZ between RBs.

*Prevalence:* Oocysts of this morphotype were found in 174/674 (26%) *M. berezovskii*, being found in 79/185 (43%) juveniles, in 42/263 (16%) adult males, and in 53/226 (23%) adult females.

*Sporulation:* Exogenous. Oocysts were shed unsporulated and became completely sporulated after 2 weeks in 2.5% (w/v) potassium dichromate solution (K_2_Cr_2_O_7_).

*Prepatent and patent periods*: Unknown.

*Site of infection:* Unknown, oocysts were recovered from feces after the animals defecated.

*Pathogeny:* Unknown.

*Materials deposited:* Photosyntypes [5] of sporulated oocysts are deposited in the Key Laboratory of Non-Invasive Research Technology for Endangered Species, School of Nature Conservation, Beijing Forestry University, Beijing, China, repository number is BFU-E-4, 2020.

*Etymology:* This species was named in honor of Kai Li, a professor of the Key Laboratory of Non-Invasive Research Technology for Endangered Species, School of Nature Conservation, Beijing Forestry University, Beijing, China, in recognition of his numerous contributions to our understanding of wildlife conservation and research.

*Remarks:* The *L*/*W* ratio of sporulated oocysts of this species is similar as the *L*/*W* ratio of *E. jinfengshanenisis*. However, many features truly distinguish them from each other including oocyst and sporocyst size ranges, the number and shape of their RBs in the SZ, and the presence of a SB in the sporocysts of *E. kaii* that the sporocysts of or absent of *E. jinfengshanenisis* lack (Tables 1, 2).

### *Eimeria oocylindrica* n. sp. (Figs. 1K, 1L; Fig. 2F)

urn:lsid:zoobank.org:act:4DC3D38E-BBB8-4A70-B98D-9CC7F150DD82

*Type host:* Artiodactyla: Moschidae: *Moschus berezovskii* Flerov, 1929, Chinese forest musk deer.

*Type locality:* Guangyuan (32°37′ N, 104°45′ E), Sichuan Province, China.

*Other hosts:* Unknown, none to date.

*Description of sporulated oocyst:* Oocyst shape: cylindroidal, ellipsoid, to elongate ovoidal; *L* × *W* (*n* = 27): 36.0 × 21.4 (33–41 × 19–26), *L*/*W* ratio: 1.7 (1.6–1.8); oocyst wall smooth and colorless to pale yellow, ~1.3 thick, bi-layered; oocyst wall thickest on sides and thinnest at end opposite M; MC: absent; M: present (*n* = 23), ~2.6 (0.8–6.7) wide; 1 or 2 obvious PG present (1.6–2.0 wide), OR absent.

*Description of sporocyst and sporozoites:* Sporocyst shape: elongate ovoidal; *L* × *W* (*n* = 25): 13.8 × 7.7 (11–16 × 6–9.5); *L*/*W* ratio: 1.8 (1.5–2.2); SB present; SSB, PSB: both absent; SR: present as small granules disbursed between SZ; SZ sausage-shaped with large RB at more rounded end and a smaller RB at the more pointed end; N, when visible, in middle of SZ between RBs.

*Prevalence:* Oocysts of this morphotype were found in 113/674 (17%) *M. berezovskii*, being found in 49/185 (26%) juveniles, in 26/263 (10%) adult males, and in 38/226 (17%) adult females.

*Sporulation:* Exogenous. Oocysts were shed unsporulated and became completely sporulated after 2 weeks in 2.5% (w/v) potassium dichromate solution (K_2_Cr_2_O_7_).

*Prepatent and patent periods*: Unknown.

*Site of infection:* Unknown, oocysts were recovered from feces after the animals defecated.

*Pathogeny:* Unknown.

*Materials deposited:* Photosyntypes [5] of sporulated oocysts are deposited in the Key Laboratory of Non-Invasive Research Technology for Endangered Species, School of Nature Conservation, Beijing Forestry University, Beijing, China, repository number is BFU-E-7, 2020.

*Etymology:* The species name is derived from oocyst shape based on oocyst and cylindr- (Gk., cylinder).

*Remarks:* The sporulated oocysts of this species are similar in shape to those of *E. jinfengshanenisis* described by Sha et al. [19] but their oocyst dimensions are very different (Table 1). The size range of *E. oocylindrica* oocyst is most similar to those of *E. dolichocystis* and *E. fengxianensis*. They differ from those of *E. dolichocystis* by having a much smaller *L*/*W* ratio (1.7 vs. 2.0), and they lack an OR which is present in oocysts of *E. dolichocystis* (Table 1). They differ from those of *E. fengxianensis* by having a different shape, a thinner oocyst wall, a larger *L*/*W* ratio (Table 1) and two RBs vs. one RB in the SZ of *E. fengxianensis* (Table 2).

Four species of *Eimeria* were already described from *Moschus berezovskii.*

### *Eimeria jinfengshanenisis* Sha, Zhang, Cai, Wang and Liu, 1994

*Type host:* Artiodactyla: Moschidae: *Moschus berezovskii* Flerov, 1929, Chinese forest musk deer.

*Remarks:* Sha et al. [19] published a short note describing two new *Eimeria* species from *M. berezovskii* they sampled in Sichuan Province, China. The oocysts they described were broadly ovoidal and measured, 28.1 × 18.3 (25–32.5 × 17.5–18.5); *L*/*W* ratio: 1.6 (sic) (1.4–1.8) with a thin, 2–layered oocyst wall, ~0.5–0.8 thick and an inconspicuous M and lacking OR and PG. Their sporocysts were ellipsoidal, 11.1 × 5.9 (10–11 × 5–7); *L*/*W* ratio: 1.9 (1.7–2.0), without SB, SSB, or PSB, but a SR of small granules between SZ was present (see Tables 1, 2 for other details). They compared the oocysts of this species to those of *E. faurei* from the domestic sheep because they thought that theirs were the first two eimerians ever found in any species of *Moschus*. Clearly, they were unaware of the paper by Matschoulsky [15] who earlier had described two eimerians from *Moschus moschiferus* L., 1758, the Siberian musk deer.

The sporulated oocysts of this species are among the smallest of all eimerians now known from *Moschus* species and clearly differs from the others in several structural features (see Tables 1, 2). One obvious structural feature that is missing in the work of Sha et al. [19] is that the sporocysts of this species lack a SB, both in their written description and line drawing. We suspect that this was a *lapsus calami* on their part since very few (true) eimerians in the Artiodactyla are reported without a SB (and some of these are also in error).

### *Eimeria moschus* Matschoulsky, 1947

*Type host:* Artiodactyla: Moschidae: *Moschus berezovskii* Flerov, 1929, Chinese forest musk deer.

*Remarks:* As far as we know, Matschoulsky [15] published the first paper identifying and (partially) describing the oocysts of two *Eimeria* parasites in musk deer, but the paper was in an obscure journal and most scholars who have summarized the coccidia known in Artiodactyla and/or in *Moschus*, as indicated by Pellérdy [17], Levine and Ivens [11, 12], Levine [10], Sha et al. [19], were unaware of or unable to obtain a copy of Matschoulsky’s in 1947 [15]. Fortunately, we have a copy of his paper in our archive and our best translation of that paper (from Russian) tells us that Matschoulsky in 1947 [15] said, “We studied feces of musk-deer, from Kunkien region, and found two different species of coccidia,” which he named *E. moschus* and *E. sajanica*. Later authors [11, 12, 17], who did not see Matschoulsky’s original paper, refer to information given by Svanbaev [22] who referred to Matchoulsky’s earlier work, did not mention *E. moschus*, and seemed to presume that *E. sajanica* was found in *Saiga tatarica* “found in Asian territory of the Soviet Union.” In 1947, Matschoulsky [15] said these oocysts from musk deer were ovoidal with a two-layered wall, a well-defined M up to 6.6 wide, and measured 27.4 × 20.9 (20–31 × 15–23); *L*/*W* ratio: 1.3; OR, PG: both absent. Sporocysts were ellipsoidal, 10–10.5 × 5–7 (means not given) and SB, SSB, PSB were all absent while SR was present as scattered small granules and SZ had one small RB at its more rounded end (line drawing). Matschoulsky [15] mentioned neither the host species name nor the specific locality from which the oocysts were collected, but he did state the two species he was describing were from a musk deer so we must presume the host was *M. moschiferus*, which is found in Siberia. The description of this species is marginal by even the most lenient current standard, but Matschoulsky [15] did provide a line drawing to illustrate the specimen he described. We believe the most prudent thing to do now is to accept his name (since it has priority) and description until future studies can confirm or dispute its existence.

### *Eimeria dujiangyanensis* (Sha et al., 1994) n. nom.

urn:lsid:zoobank.org:act:8E81F0A4-9E6A-4AB1-ABC6-FBD13CC54963

*Synonym: Eimeria moschus* Sha, Zhang, Cai, Wang and Liu 1994.

*Type host:* Artiodactyla: Moschidae: *Moschus berezovskii* Flerov, 1929, Chinese forest musk deer.

*Remarks:* Sha et al. [19] did not read Matschoulsky’s [15] paper using the same name (*Eimeria moschus*) for an eimerian they described from *M. berezovskii* in China. Their oocysts had ovoidal sporulated oocysts that measured, 35.2 × 26.9 (32.5–37.5 × 25–30); *L*/*W* ratio: 1.3; M: present, ~6–7 wide; OR, PG: both absent. Their sporocysts were spindle-shaped, pointed at both ends, 17.5 × 8.2 (16–20 × 7.5–10); *L*/*W* ratio: 2.1 (1.9–2.3); SB, SSB, PSB: all absent; SR: present as scattered small granules between SZ; SZ: with one RB at its more rounded end (line drawing).

*Etymology:* We propose a new name for the species to avoid synonymy. The new species name is derived from the name of the location within Sichuan Province where Sha et al. (1994) found this species and -*ensis* (Latin, belonging to).

### *Eimeria sajanica* Matschoulsky, 1947

*Type host:* Artiodactyla: Moschidae: *Moschus moschiferus* L., 1758, Siberian musk deer.

*Remarks:* Matschoulsky [15] published the first paper identifying and (partially) describing two eimerian parasites in musk deer but the paper was in an obscure journal and most scholars who have summarized the coccidia known in Artiodactyles and/or in *Moschus*, as indicated by Pellérdy [17], Levine and Ivens [11, 12], Levine [10], Sha et al. [19], either were unaware of, or were unable to obtain a copy of his paper in 1947 [15]. Our translation (from Russian) of that original paper [15] is: “We studied feces of musk-deer, from Kunkien region, and found two different species of coccidia,” which he named *E. moschus* and *E. sajanica*. Later authors, as noted above, referred to information given by Svanbaev [22], who mentioned Matchoulsky’s work [15], but presumed that *E. sajanica* was found in *Saiga tatarica* “in Asian territory of the Soviet Union.” However, if we trust that Matschoulsky [15] said these oocysts were from musk deer, then they must be from *M. moschiferus*, the Siberian musk deer. Matschoulsky [15] said these oocysts from the musk deer were ovoidal to round with a two-layered wall, but lacked a M, and measured 20.7 × 18.3 (18–23 × 16.5–20); *L*/*W* ratio: 1.1; OR, PG: both absent. Sporocysts were ellipsoidal, 5–10 × 3–5 (means not given); SB, SSB, PSB: all absent; SR: present as scattered small granules between SZ; SZ: with one small RB at more rounded end (line drawing). The description of this species is marginal by even the most lenient current standard, but Matschoulsky [15] provided a line drawing to illustrate the specimen he described. We believe the most prudent thing to do now is to accept his name and description until future studies can confirm or dispute its existence.

## Discussion

In summary, 10 species of *Eimeria* are now known to parasitize two of the seven extant *Moschus* species; the remaining five *Moschus* species have never been examined for coccidian oocysts. These 10 coccidians include the six new species described here from *M. berezovskii* (*E. aquae, E. dolichocystis, E. fengxianensis, E. helini, E. kaii*, *E. oocylindrica*), two species described by Sha et al. [19], also from *M. berezovskii* (*E. dujiangyanensis, E. jinfengshanenisis*), and the first two species that were (partially) described by Matschoulsky [15] from *M. moschiferus* (*E. moschus, E. sajanica*). This is the first comprehensive report of the coccidian species known from all forest musk deer (*Moschus* spp.).

Our study demonstrated a high prevalence (65%) of six *Eimeria* species in the forest musk deer we sampled, especially in juveniles (85%), which is similar to infection rates found by other workers in China [2, 3]. The infection prevalence of *Eimeria* between female and male adults was not significantly different in our research, which had been observed in Cai et al. [2], when they compared the prevalence of *Eimeria* oocysts between males (46%) and females (45%) in 2016, and did not find any difference in genders (*p* > 0.05). In all fecal samples, *E. aquae* was the most prevalent species (38%, 255/674), while the other five eimerians we discovered have lower prevalence in these endangered deer: *E. kaii* 26% (174/674), *E. helini* 24% (159/674), *E. oocylindrica* 17% (113/674), *E. dolichocystis* 11% (73/674), and *E. fengxianensis* 7% (45/674). Of all positive samples, 60% were infected with multiple *Eimeria* species. These results suggest to us that *Eimeria* spp. in these musk deer may be the main risk factor in limiting their health and contribute as the leading negative effect on juvenile musk deer survival. We suggest that integrated strategies are essential to prevent and/or at least control coccidial infection in these populations.

Unfortunately, this is the sum total of our knowledge about the biology of all species in the Eimeriidae, now known to infect musk deer. We know nothing about any of their endogenous stages (merogony and the number of generations, merozoites produced at each stage, micro- and macrogametocytes, fertilization, oocyst wall formation) or the cells and locations of these stages in infected cells or even within the gastrointestinal tract (or elsewhere?); we know nothing about the pathology each of these eimerians may cause in musk deer and whether some are more pathogenic than others; we know nothing about how long these eimerians can persist within their hosts (prepatent and patent periods); there have not yet been any cross-transmission studies with any of the 10 known *Eimeria* species so we do not know if each can infect other wild or domestic Artiodactyla; we know nothing about the host’s immune response to each of these eimerians or if previous infection produces lasting or only transient immunity to that species or even to other species; we do know that many of the 10 known eimerians have sporulated oocysts that share many similarities, but there have not yet been any genes sequenced of any of these oocysts and, thus, no phylogenetic analyses have been possible to determine whether or not these oocysts, or certain combinations thereof, represent 10 or perhaps fewer real species. Clearly, we are only at the discovery stage and there is a lot more work to be done before we can gain any real understanding of the biology and consequences of *Eimeria* species infections in our endangered and protected musk deer.

## Conclusion

In captivity, the confined areas and high stress would accelerate host susceptibility to parasites. Additionally, coccidiosis caused by *Eimeria* leads to severe enteric disease in captive musk deer, while little is known about these species, even for their classification. To the best of our knowledge, our work is the first comprehensive report on the coccidian species known from all musk deer. We summarized 10 species of *Eimeria* known to parasitize musk deer, including six new species: *E. helini*, *E. huangi*, *E. oocylindrica*, *E. shengi*, *E. dolichocystis*, *E. kaii*, Our research also demonstrated a high prevalence of infection in forest musk deer, usually with multiple *Eimeria* species. Thus, we argue that coccidia in the wildlife, especially for endangered animals in captivity, should be identified more deeply.
